# Correction: A lab-on-a-chip for preconcentration of bacteria and nucleic acid extraction

**DOI:** 10.1039/c8ra90058b

**Published:** 2018-07-27

**Authors:** M. Hügle, G. Dame, O. Behrmann, R. Rietzel, D. Karthe, F. T. Hufert, G. A. Urban

**Affiliations:** Laboratory for Sensors, Department of Microsystems Engineering (IMTEK), University of Freiburg Georges-Köhler-Allee 103 Freiburg Germany; Institute of Microbiology and Virology, Brandenburg Medical School Theodor Fontane Neuruppin Germany; Helmholtz-Centre for Environmental Research Magdeburg Germany; German-Mongolian Institute of Resources and Technology Nalaikh Mongolia

## Abstract

Correction for ‘A lab-on-a-chip for preconcentration of bacteria and nucleic acid extraction’ by M. Hügle *et al.*, *RSC Adv.*, 2018, **8**, 20124–20130.

The authors regret that affiliation details for the author Daniel Karthe are missing from the original article. The correct affiliation details are shown here.

The Royal Society of Chemistry apologises for these errors and any consequent inconvenience to authors and readers.

## Supplementary Material

